# National capacity strengthening within the context of an international vector control partnership: findings from a qualitative study conducted within the Ugandan ‘Tiny Targets’ programme

**DOI:** 10.1136/bmjph-2023-000410

**Published:** 2024-04-22

**Authors:** Siya Aggrey, Justin Pulford, John Bosco Bahungirehe, Charles Wamboga, Andrew Hope

**Affiliations:** 1 Liverpool School of Tropical Medicine, Liverpool, UK; 2 Coordinating Office for Control of Trypanosomiasis in Uganda, Kampala, Uganda; 3 Ministry of Health, Kampala, Uganda

**Keywords:** Public Health, Disease Eradication, Endemic Diseases

## Abstract

**Introduction:**

The Ugandan Tiny Target programme is an example of an international vector control partnership that held specific capacity strengthening objectives in support of a disease elimination goal. Drawing on this experience, we sought to derive transferable lessons that may inform capacity strengthening approaches within other partnership-based vector control programmes.

**Methods:**

A longitudinal qualitative study encompassing semistructured interviews conducted with Ugandan partners working on the Tiny Target programme. Data analysis was informed by a general inductive approach.

**Results:**

Capacity strengthening priorities evolved over time initially focusing on the immediate capacities needed to perform roles and responsibilities assigned within the partnership and then shifting towards more advanced, transferable knowledge and skills. A distinction between operational and systemic priorities was observed: the former was necessary to support successful programme implementation whereas the latter reflected fundamental limitations or complexities within the Ugandan context that were bypassed by including an international partner. Systemic priorities were fewer in number than their operational counterparts, although substantially harder to resolve. The largest apparent threat to the long-term sustainability of reported capacity gains was their concentration within a small number of individuals.

**Conclusion:**

Our study highlights three key lessons that may inform the design of national capacity strengthening activities conducted within the context of international vector control partnerships, including (1) Multiple approaches to strengthen capacity are needed and that can adapt to changing capacity strengthening priorities over time; (2) Balancing operational and systemic capacity strengthening priorities, the latter becoming increasingly important within longer-term partnerships and (3) Partnership members in focal country/ies should be supported to actively facilitate the transfer of newly acquired knowledge and skills to relevant colleagues/communities outside of the partnership. The generic nature of these recommendations suggests they are likely to be of benefit to many and diverse international partnerships within the wider global health space.

**WHAT IS ALREADY KNOWN ON THIS TOPIC**

- Strengthening vector control capacity is a foundation of the WHO’s global vector control response, 2017–2030.
- International vector control partnerships are one means by which vector control capabilities can be strengthened in less well capacitated countries. However, there are few research-derived accounts of how capacity strengthening opportunities can be optimised within the context of such partnerships.

**WHAT THIS STUDY ADDS**

- We provide pragmatic, research-derived recommendations for designing and implementing capacity strengthening activities within international vector control partnerships.

**HOW THIS STUDY MIGHT AFFECT RESEARCH, PRACTICE OR POLICY**

- International vector control partnerships, as well as bodies that fund such partnerships, can draw on these recommendations to inform more impactful capacity strengthening practice. As the recommendations are relatively generic in nature, they are also likely to be of value to a broad range of partnerships working in the global health space.

## Introduction

 Human African trypanosomiasis (HAT), also known as sleeping sickness, is caused by two parasites, *Trypanosoma brucei gambiense* and *Trypanosoma brucei rhodesiense*, transmitted by tsetse flies and is generally fatal if not treated. The *Gambiense* form of the disease (gHAT) accounts for over 90% of cases.^1^ There were three major gHAT epidemics in the 20th century, the most recent occurring in the 1990s when over 35 000 cases were reported in some years.^2^ This led to a concerted international effort, coordinated by the WHO, to bring the disease under control. These efforts brought the case numbers down to below 10 000 per year by 2009.^3^ In 2012, WHO targeted HAT for elimination as a public health problem by 2020,^4^ defined as fewer than 2000 new cases reported annually and a 90% reduction in the areas at moderate or higher risk compared with 2000–2004.^5^ The first target was reached in 2017 and has been sustained while the second target was narrowly missed.^2^ A new 2021–2030 roadmap has been published where gHAT is targeted for elimination of transmission.^6^

Historically, control of gHAT has primarily relied on the detection and treatment of human cases.^7^ However, the role of vector control in gHAT elimination efforts has assumed greater prominence in recent years following the development of a cost-effective vector control technology, ‘Tiny Targets’. Tiny targets are insecticide-impregnated screens comprising a blue panel and a black mesh panel; the colour attracts tsetse which takes up insecticide on contact and is subsequently killed. Tiny Targets are cheap, logistically easy to deploy and have been shown to reduce tsetse densities by >80%; modelling has demonstrated that a sustained 72% reduction of tsetse populations will stop HAT transmission.^8^,^13^ Analyses have shown that Tiny Target interventions significantly reduce the incidence of gHAT, thus demonstrating the value of adding vector control to screening and treatment efforts.^9 11 14^

Tiny Target implementation in gHAT endemic countries has taken place within the frame of international partnerships. For example, the Liverpool School of Tropical Medicine (LSTM) based in the UK has partnered with relevant national organisations in Cameroon, the Democratic Republic of Congo and Uganda in support of Tiny Target-based vector control programmes.^12 13 15^ International partnerships of this type can be highly successful with respect to the underlying disease control, prevention or elimination objective/s. As a case in point, Uganda received validation from WHO in 2022 that gHAT has been eliminated as a public health problem, driven in large part by international partnerships focused on supporting both case detection and treatment and vector control.^16 17^ International partnerships can also enhance vector control capacity and capability in less well capacitated partner countries,^18^ a foundation of the WHO’s global vector control response, 2017–2030.^19^ Despite this potential, there are few research-derived accounts in the published literature on how capacity strengthening components embedded within international vector control partnerships function or perform. Research of this type is needed to ensure capacity strengthening practices within such partnerships are optimised.

In this paper, we present findings from a qualitative study conducted in support of national capacity strengthening within the ‘Targeting Tsetse’ vector control project. Targeting Tsetse was an international partnership between LSTM and the Coordinating Office for Control of Trypanosomiasis in Uganda (COCTU). This partnership formally commenced in 2014 with the primary objective of implementing a Tiny Target programme in support of Uganda’s gHAT elimination goal. The partnership further supported national capacity strengthening in vector control operations through the provision of technical assistance, a ‘learn-by-doing’ implementation model and via a bespoke capacity strengthening action cycle (described further below). Overarching aims of these capacity strengthening activities were to ensure national partners were sufficiently capacitated to implement the Tiny Target programme as intended and to facilitate a shift towards greater national independence in tsetse control, that is, less reliance on international partners to implement tsetse control as needed. Our study had multiple objectives, including (1) identifying the capacity strengthening priorities of the Ugandan partners in the project; (2) exploring the project-specific capacity strengthening processes, experiences and outcomes; (3) informing future directions within the partnership and (4) deriving transferable lessons that may inform national capacity strengthening approaches within other partnership-based vector control programmes.

Capacity strengthening is often not defined when discussed in the literature and standardised definitions have not been widely agreed.^20^ However, contemporary descriptions typically refer to three levels of capacity strengthening, including the individual, the organisational and the societal (or national), emphasising a ‘whole system’ approach to capacity strengthening in the focal area.^21 22^ Terminology also varies with the phrasing ‘capacity strengthening’, ‘capacity building’ and ‘capacity development’ often used interchangeably,^20^ despite efforts to differentiate between the terms.^23^ Traditional notions of capacity strengthening have been critiqued in the Global Health literature on the grounds that they perpetuate assumptions of ‘Northern’ superiority over ‘Southern’ counterparts and/or Southern knowledge systems.^24^ As such, the notion of bidirectionality or multidirectionality in capacity strengthening which recognises all members of any partnership, irrespective of their respective location or existing capacities, gain new knowledge, skills or experience as a result of the partnership is increasingly promoted^22^; although recognising that within an equitable partnership, some members may require additional capacity strengthening support as compared with others. While our study focuses solely on the capacity strengthening process, experience and outcomes of Ugandan partners within a UK–Uganda partnership, we wholeheartedly recognise that the capacity gains were bidirectional. Our focus on the Ugandan partner experience within this paper is primarily informed by the explicit call from the WHO to build vector control capacity within the Global South^19^ and recognition that international, disease control and elimination partnerships of which the Tiny Target is just one of multiple examples can afford an excellent opportunity to do so if appropriately designed and delivered.

## Method

### Study design

We employed a highly pragmatic, operational research design as our overarching aim in undertaking this study was to inform capacity strengthening activities that could be delivered at mid and late stages of the Tiny Target partnership (following the first and second rounds of interviews, respectively).

### Study setting

Targeting Tsetse was initially a 4-year project with funding to implement tsetse control over a 2500 km^2^ area in five districts (Arua, Koboko, Maracha, Moyo, Yumbe) of NW Uganda working through entomologists employed in the respective district administrations. During the first year of the project, LSTM was responsible for coordinating activities (sensitisation, biannual deployment of targets, quarterly entomological monitoring) with the district entomologists across the five districts and COCTU provided some on-the-ground supervision and was involved in some aspects of training. From late 2015, COCTU assumed the lead role for coordinating project implementation with LSTM taking a supporting role. Since the beginning of the project, tsetse control has been successfully implemented across the intervention area with multiple rounds of Tiny Target deployment and results from entomological monitoring demonstrating impact on tsetse populations.^13^ Operations were further expanded with additional funding awarded in 2017 under the Trypa-NO! grant that led to a further 2500 km^2^ of control including introduction of Tiny Targets in two new districts as well as expansion in pre-existing intervention areas. Trypa-NO! was renewed in 2020 with tsetse control scale-back introduced during this phase and a subsequent renewal for 2023–2025 will see a complete withdrawal of Tiny Targets and a shift in focus to postelimination monitoring.

Capacity strengthening activities initially focused on imparting the technical and operational skills and knowledge necessary for successful programme implementation and to support the phased transition between LSTM and COCTU leadership. This included such things as training on how to assemble and deploy Tiny Targets, training on GPS use and GPS data management which were variously directed towards COCTU staff with programme responsibilities, district entomologists engaged in the programme and the deployment teams who worked under their direction. In late 2017, by which time COCTU and district staff had successfully deployed Tiny Targets for multiple rounds, a complementary capacity strengthening ‘action cycle’ was introduced. Based on the five-step approach to capacity strengthening,^25^ the action cycle was designed to identify and address remaining capacity gaps as prioritised by COCTU staff and the district entomologists with a view towards ensuring programme sustainability and greater national independence.

### Sampling

All district entomologists tasked with leading deployment of the Tiny Target programme in their respective district and all COCTU staff with assigned programme responsibilities were included in this study.

### Procedure

All data were collected prospectively via semistructured interviews (SSIs) completed at two time points, November 2017 and November 2022. The Tiny Target programme was operating at maximum scale in all seven districts in the first of these time periods and had been scaled back in four districts by the second. An introductory email outlining the aims of the study and with the formal information sheet attached was sent to all prospective participants at least 2 weeks prior to interview on both occasions. The email text and the information sheet clearly stated that participation was voluntary and that a decision not to participate would not affect their involvement in the Tiny Target programme in any way. Written informed consent was obtained prior to participation in all cases. All interviews were conducted in-person at the workplace of the respective participant during regular working hours and followed a topic guide tailored to the role of the participant and/or the stage of data collection (online supplemental file 1). In the first round of interviews, the topic guide primarily focused on challenges experienced in programme implementation to date, threats to sustainability and remaining capacity gaps. During the second round, the topic guide primarily focused on experience and outcomes of the capacity strengthening process to date, ongoing threats to sustainability and future partnership aspirations. All interviews were conducted in English, were audio recorded, typically lasted between 45 and 60 min and were subsequently transcribed in full. Field notes were taken at the time of interview by one of the two lead interviewers (by turn).

### Reflexivity statement

The interviews were conducted by the lead authors (JP and SA), who hold PhD and master’s degrees, respectively. The lead interviewer (JP) was a white male independent of all operational aspects of the Tiny Target programme, although was an LSTM employee himself. The colead interviewer (SA) was a male Ugandan contracted by LSTM to support this study as well as the development of programme-specific information, education and communication resources. Given both lead interviewers had some, although minor, affiliation with the Tiny Target programme and given one lead interviewer was Ugandan, it is unclear as to whether participants afforded them ‘insider’ or ‘outsider’ status. This ambiguity may have influenced participant responses, although the degree and type of any such influence would likely have varied across participants (depending on the conclusions each participant drew regarding the interviewers’ positionality). All except two participants were male, suggesting any gender-related biases resulting from a lack of female representation within the interview team were likely to have been minimal. Both lead interviewers have several years’ experience conducting interviews within the frame of qualitative research in Uganda (SA) and throughout sub-Saharan Africa (JP) and codeveloped and piloted the topic guides prior to use. These experiences undoubtedly influenced the wording and style of participant interviews, which were open yet somewhat formal and intended to convey respect for their contribution to the Tiny Target programme and the professional district or government positions in which all were employed. Tiny Target project management staff from both LSTM (AH) and COCTU (JBB) were present for most interviews. We recognise the presence of these project management staff may have biased participant responses towards more positive or less contentious comments. However, participants were repeatedly reassured that they could provide open and honest answers without fear of consequence, and all understood that their responses (especially during stage one interviews) would directly inform capacity strengthening activities within the partnership. Thus, there was a clear incentive to explicitly acknowledge any challenges and capacity gaps experienced.

### Data analysis

All interview transcripts were uploaded into NVivo software (V.12). Transcripts were not provided to participants for comment or correction, although workshops were held with participants at the end of each interview round in which formative summaries of the collected data were discussed. Data analysis was informed by a general inductive approach,^26^ aligning emerging themes identified in the data with predetermined focal areas relevant to the overarching study objectives. Transcripts were initially coded by the lead author (JP), resulting in a draft data framework. A second author (SA) then conducted a second round of coding against this framework. The subsequent framework, including primary themes, subthemes and a draft narrative, was then critically reviewed by three coauthors (JP, AH and SA). Final coding decisions were agreed upon consensus opinion.

### Patient and public involvement

No patients or members of the public were involved in the design, conduct or reporting of this study.

## Results

### Participation

11 SSIs were completed at each interview round, resulting in a total of 22 SSIs completed with 14 individuals, 8 of whom were interviewed at both time points. Where the same individual was not interviewed twice, this was due to a change in staffing over the course of the project. No one invited to interview declined to participate. In total, 14/22 interviews were completed with district entomologists and 8/22 with COCTU staff. Participant quotes presented below have been anonymised. The codes ‘DE’ and ‘C’ refer to district entomologist and COCTU staff, respectively. Codes were individualised, so the same code is always applied to a quote from the same individual, with the letter ‘b’ indicating the quote was taken from the second interview with the same person.

### Capacity strengthening priorities

Participants identified a wide range of capacity strengthening priorities, which were broadly categorised into two types: ‘operational’ and ‘systemic’. Operational priorities were categorised as such as they generally pertained to the assigned Tiny Target programme responsibilities of COCTU or district staff. Most of these operational priorities were identified in the first round of interviewing with many presenting as essential to address to ensure successful or safe programme implementation such as operating a global positioning system (GPS) unit, creating maps using geographical information system (GIS) software or training on the management of field risks. Operational priorities identified in the second round of interviewing were typically less essential, in the sense that they were not necessary for ongoing programme implementation (or safety), yet participants recognised the value in gaining greater proficiency in these areas. For example, one participant was keen to learn more advanced functions on their GPS unit that had not been needed for Tiny Target deployment:

I have been training the deployers on GPS. On that, GPS has so many functions. Some of them I’ve not exploited. I’ve not entered into those, some of the functions. (DE1b)

Systemic capacity strengthening priorities, on the other hand, in no way impacted the implementation of the Tiny Target programme as currently operationalised. That is, they did not need to be resolved to achieve the primary objective of eliminating gHAT as a public health problem in Uganda within a partnership model. Rather, systemic priorities reflected fundamental limitations or complexities within the Ugandan context that were circumvented by the inclusion of an international partner. In other words, because of capacity constraints in these areas, COCTU and the Ugandan district entomologists were unable to implement the Tiny Target programme as conceived independently. Systemic priorities were far fewer in number than their operational counterparts, although they presented as being substantially harder to resolve. Key systemic capacity gaps included the supply of Tiny Targets which were procured internationally by LSTM, complex financial management and procurement bureaucracies as well as limited programme funding:

The reason why COCTU would go out to partner with development partners to help these activities is the fact that the funds for entomological work are not there [within Uganda]. Some of these things, we are undressing before you. You know very well it is not enough. Each time these funds are brought [by development partners], including the district officials, they are happy that at least something is on the ground going to help the population. That is the truth. There are no funds. (C2)

### Capacity strengthening experience

All participants reported capacity gains stemming from their involvement in the Tiny Target programme. Most of these gains were ‘operational’ in nature and most were realised at the level of the individual, as illustrated by the following quote:

I think this project has really exposed me also, first of all, knowing the GPS, how to operate. Administratively it has helped me, so that I’m able to manage the deployers in terms of administration. And the project has also made me to be the person to account, make use of funds as intended…and the accountabilities are all complete, meaning that it has built my potential of being able to account for the resources. (DE8b)

There were comparatively fewer examples of capacity strengthening within the ‘systemic’ priority areas. Programme funding and complex bureaucracies were considered equally problematic at the time of the second interview as they were at the first, although some initial steps had been taken regarding Tiny Target supply as indicated in the following quote:

The government provided some seeding money to train a team of 21 youth and women who were trained for about two weeks. They were provided with the skills to manufacture these Tiny Targets as a trial. Throughout that process they could have made about 20 targets. But what disturbs the whole arrangement about the Tiny Target is the knowledge that [name of commercial manufacturer] has put in place in terms of how they manufacture the Tiny Target. Because we have been told that the cloth is factory-treated, that the chemical is within. This is what complicates the process. Even if we made a target using the normal materials, how long are we able to keep the active ingredient in that piece of cloth? (C1b)

Capacity gains were not uniform across participants, with variance in the number, type and magnitude of gains reported. Some of this variance is related to the different programme responsibilities, and therefore different learning opportunities and capacity strengthening priorities, between participant groups. In other cases, the variance reflected the different ‘starting points’ of participants, even among those with the same role and responsibilities. One district entomologist, for example, revealed that his participation in the Tiny Target programme represented his first-ever opportunity to use a computer:

The knowledge that I’ve got on computers has really helped me a lot. That is one very important thing that I’ve got from the project, yes, the knowledge on computers. Because at the beginning, if the project was not even coming, I may not have even taken the initiative to learn computers*.* (DE7b)

The reported capacity gains were facilitated by multiple mechanisms. Proficiency and new capacities in most routine programme activities were typically gained relatively early via learn-by-doing approaches, with subsequent capacity gains primarily facilitated through formalised ‘action cycle’ activities. Constructive feedback, from LSTM to COCTU, and from COCTU to district entomologists, over the duration of the programme was also identified as a capacity strengthening mechanism, especially in relation to the preparation of detailed budgets and programmatic/technical reports. Mentorship also played a role in some cases as did peer support. Illustrative examples of two mechanisms, learn-by-doing and constructive feedback, are presented below:

Most of the improvements [in own capacity] have been operational. You pick up things as you move along. (C1b)

We have not been doing things in isolation. Many times, I’ve called [name of COCTU staff member] in Kampala. I’m looking at this, can you please help me here? I’m a bit stuck. Yes, that’s how we have been doing, how we have been succeeding in our work. We consult, a lot. (DE7b)

Clear threats to the sustainability of reported capacity gains were evident. The biggest threat was the apparent concentration, at district level, of essential knowledge and skills within a single person: the district entomologist. If this person leaves their district post or is incapacitated in some way, then the loss of experience and knowledge at district level may not be easy to replace:

You remember I’m alone in the district, in the office of the District Entomologist. I’m just alone as I talk right now. I have no assistant. (DE7)

In some cases, district entomologists who had experienced Tiny Target scale back at the time of the second interview also reported that they were beginning to forget knowledge or skills acquired during the programme:

I tried to make maps, but after the scale-down, I’m not doing any practising. Somehow, you’ll find you start forgetting the steps. (DE2b)

Some steps had been taken to mitigate the risk of losing knowledge through the departure of a staff member or lack of practice, including the provision of programme manuals and job aides at district level, although these had been misplaced in some instances and in other instances participants were unaware of their existence as illustrated below:

We don’t have any manuals. I think even the DE before he died I don’t think he had any manuals given to him. So, I think we need manuals for the Tiny Target work*.* (DE3) (Nb. Manuals and job aides had been provided to this district office).

However, there were examples of programme-acquired capacities being embedded within organisational structures or being applied outside of the Tiny Target programme for different purposes (examples below). Reported capacity gains are more likely to be sustained in these cases.

When you look at the entire management at COCTU level, we have had small but meaningful things that have been changing. Those are all dependent on the comments that come up in terms of improvement from LSTM. (C1b)

I’ve used the GIS [resources and training supplied by the Tiny Target programme] for locating beekeepers and beehive sites [in the district]. Now, all their sites are referenced. It has been very useful. And when I submit reports to the Ministry, they always feel happy. In fact, I was cited as one of the staff who gives accurate data on the locations of beehive sites in the district. (DE2b)

There were also some examples of participants transferring knowledge and skills acquired during the programme to colleagues outside of the Tiny Target programme, although many participants lacked the confidence to do this. For example, when asked if they felt able to provide the training and knowledge acquired through participation in the Tiny Target programme to other staff in their district office, one participant replied:

I could be more confident when I’m trained a second time. This is what I was thinking, so that I pick the missing gaps. I cannot say that now I’m confident, until I pick the missing gaps. (DE1b)

### Future directions

Without exception, all participants described their involvement in the Tiny Target programme in positive terms and expressed a desire for continued partnership beyond the current project end date. Underlying this positive appraisal was a mix of pride in what the Tiny Target programme had achieved, broad experience of the partnership as respectful, equitable and mutually beneficial and a recognition that the systemic issues such as limited national funding for programme implementation (as noted in the quote below) that the partnership had been able to circumvent remained problematic.

Getting independent funding is not something that is obvious. If you got independent funding somehow, you will need the technical support. Yes, one may say the time must come when you should be independent, but we’re saying that it’s not advisable to have a quick breakaway. (C1b)

Having achieved the primary programme objective, WHO-accredited elimination of gHAT as a public health problem in Uganda, there were mixed opinions as to what form an ongoing partnership might look like. Many participants raised concerns that communities would be unhappy to lose access to the Tiny Targets as they were effective in reducing tsetse numbers. The corresponding reduction in biting nuisance was perceived to be highly valued by community members and may even have been considered the primary benefit of programme participation for many, given the relative scarcity of sleeping sickness even at the time of programme onset. Thus, there was strong interest in continuing to deploy Tiny Targets as a tsetse control measure independent of a disease control objective:

When we move to the villages especially at the water points they tell us they used to be bitten so much by flies when they were washing along the river, but now it has reduced, and they keep thanking us for the activities*.* (DE2)

Nevertheless, the potential for new partnership activities beyond the continuation of a Tiny Target programme was also recognised:

This partnership was started with the programme of Tiny Targets and the elimination of sleeping sickness in West Nile, the one I have with LSTM. But I’m also aware that LSTM has capacity beyond the partnership we are having So, looking forward, I want this true partnership which we have piloted. To me, on behalf of [COCTU], I regard it as hugely successful. (C7)

## Discussion

Our study examined the capacity strengthening process that took place within the context of an international vector control partnership, from the perspective of the national partners located within the intervention country, Uganda. Our findings revealed that many of the capacity strengthening priorities of the national partners evolved over time; initially focusing on the immediate capacities needed to perform roles and responsibilities assigned within the partnership and then shifting towards more advanced, transferable knowledge and skills even though such capacities were not strictly needed for successful programme implementation. A distinction between operational and systemic priorities was also observed, with the former more numerous, primarily focused on individual skills and knowledge, often necessary to support successful programme implementation, yet also often relatively easy to address and, indeed, were the most cited examples of capacity strengthening gains obtained because of programme participation. Systemic capacity strengthening priorities on the other hand were fewer in number, typically non-essential to programme objectives and presented as manifestly harder to resolve and there was limited evidence of capacity strengthening in these priority areas over the partnership duration. However, without shifts in systemic capacity then the national partners will often remain reliant on international partners to meet national vector control objectives, even when operational capacities are similarly advanced. This finding sits within the broader critique of ‘vertical’ programme investment and the view that gains achieved via disease-specific programmes may be unsustainable in the absence of broader health systems strengthening.^27^ The ‘systemic’ capacity strengthening priorities were also ‘organisational’ and ‘societal’ in nature, within the three-level capacity strengthening definitions currently favoured,^21 22^ further highlighting the need for whole system approaches to capacity strengthening.

Threats to the sustainability of commonly reported capacity gains were evident. At district level, with only a few exceptions, the full range of acquired operational knowledge and skills needed to support Tiny Target deployment were concentrated within one individual: the district entomologist. The structure of the Tiny Target partnership ensured district entomologists were well connected with their regional counterparts, and written materials and job aides were provided to each district to ensure operational guidance was accessible on-site. Nevertheless, examples of these written materials being misplaced were reported and the current support structures may prove unsustainable outside of a project partnership context. One participant also acknowledged that their acquired skills were waning because of large-scale programme scale back in their district. These findings further reinforce the need for longer-term evaluation of capacity strengthening initiatives, as positive short-term outcomes may not always be sustained.^28^ Encouragingly, there were unfacilitated examples of study participants transferring their newly acquired knowledge and skills to either other, unrelated tasks (such as mapping the location of smallholder beehives in the district) or to other staff within their districts who were not involved in the Tiny Target programme. These examples were exceptional, and many participants expressed a lack of confidence in their ability to do likewise without further support. Yet the fact that such examples were evident highlights the potential to support facilitated transfer of knowledge and skills as a sustainability mechanism. Ensuring knowledge, skills and resources provided through international partnerships are widely accessible, including to individuals and organisations not formally belonging to the partnership, is also increasingly being recognised as good capacity strengthening practice.^29^,^31^ Our findings further suggest that multiple capacity strengthening approaches may be needed to support continuous capacity strengthening across long-term partnerships. Learn-by-doing approaches may be particularly effective in the early stages of a partnership, but unless roles and responsibilities change with time, capacity gains are likely to plateau relatively quickly via this approach. Training targeting evolving priorities and strong peer-support and constructive feedback processes will likely extend, complement and consolidate capacity gains.

The primary objective of the Tiny Target programme, to contribute to the elimination of gHAT as a public health problem in Uganda, was achieved just prior to the second round of interviews for this study. However, without exception, study participants did not want the programme partnership to end. There was still a strong interest at district level in maintaining Tiny Target deployment as a form of vector control, to reduce biting nuisance rather than gHAT elimination and there was an appetite to extend the partnership into new areas. On one hand, this reflects the positive experience most participants reported in relation to their partnership involvement. Consistent with good principles of global health partnership,^32^ both COCTU and district staff were empowered through the partnership to take on ever greater responsibility and the programme’s success was a point of pride for all. On the other hand, interest in continued partnership was also partly based on the recognition that without an international partner, systemic issues pertaining to funding and financial management would severely restrict the ability of both COCTU and district entomologists to continue to undertake substantive vector control activities. This reality further highlights the need to focus on systemic capacity strengthening even when not strictly necessary to do so to achieve partnership objectives as well as the benefit of longer-term partnerships. However, the benefit of longer-term partnership will only be fully realised if the partnership is used, at least in part, as a vehicle to address systemic constraints to independent practice. Without the latter focus, then the consequence to the less well capacitated partner in terms of the ‘loss’ in their ability to engage in vector control will be as great at the end of a longer-term partnership as it would be at the end of a shorter-term variant.

Among other objectives, our study sought to derive transferable lessons that may inform national capacity strengthening approaches within other partnership-based vector control programmes. Box 1 outlines three such lessons along with the study evidence and underlying logic supporting each lesson. To be effective, the recommendations inherent in all three of these lessons require the support of programme funders, programme implementers and programme participants. For example, funders must be willing to support sophisticated and multilevel capacity strengthening activities within the scope of the respective programme, even when there is no immediate or direct benefit within the programme lifespan, implementing partners must be willing to adopt and promote these activities and participants must be willing to take them on, even when it requires additional effort to do so (such as transferring skills to colleagues outside of the programme).

Box 1 **Transferable lessons for national capacity strengthening within partnership-based vector control programmes**

**Lesson 1: Capacity strengthening programmes/activities within international partnerships should consist of multiple approaches, some of which may be sequentially delivered, that can adapt to changing capacity strengthening priorities over time.**

Study evidence supporting this recommendation:

- Multiple mechanisms of capacity strengthening were identified in our study, including learn-by-doing approaches, ‘action cycles’, constructive feedback, mentorship and peer support.
- Capacity strengthening priorities evolved over time, shifting from an initial focus on essential capacities needed for programme delivery towards more advanced, highly transferable skills.

Underlying logic:

- Different approaches towards capacity strengthening, some delivered consistently (eg, mentorship, peer support), others introduced strategically at distinct time points (eg, learn-by-doing, action cycles), will allow continuous capacity strengthening across the duration of a partnership and will allow emerging capacity strengthening priorities and opportunities to be identified and addressed.

**Lesson 2: A balance should be sought between operational and systemic capacity strengthening priorities, the latter becoming increasingly important within longer-term partnerships.**

Study evidence supporting this recommendation:

- Operational capacity strengthening priorities were more commonly identified, were essential to programme success and were more readily achieved compared with systemic capacity strengthening priorities.
- Failure to address the latter resulted in a continued dependence by national organisations on their international partners for programme continuation.

Underlying logic:

- Strengthening capacities essential for programme implementation is a necessary and reasonable focus, especially in the early stages of any international vector control partnership. However, a sole focus on capacities essential for programme implementation will lead to a plateau in capacity gains over time and may do little to address the broader systemic challenges that require national organisations in the Global South to seek international assistance in the first place.

Lesson 3: Partnership members in the focal country/ies should be supported to actively facilitate the transfer of newly acquired knowledge and skills to relevant colleagues/communities who do not belong to the partnership.

Study evidence supporting this recommendation:

- Examples of non-mandated transfer of knowledge and skills acquired from partnership participation for new activities and to a broader audience demonstrated the opportunity and potential to do so.
- Substantial reluctance, in large part due to a lack of confidence, on most partnership members to transfer knowledge and skills for new activities or to a broader audience.

Underlying logic:

- Broader transfer of knowledge and skills acquired through partnership activities will add value to any programme investment and will help sustain the newly acquired knowledge and skills beyond the partnership duration.

Our study had many strengths including the longitudinal study design, the inclusion of all participating district entomologists and COCTU staff in the study sample and robust quality assurance processes. Study limitations were also present. Data were drawn from a single vector control programme in a single country context. We didn’t examine the capacity strengthening experience of the UK-based partners, who would have undoubtedly gained some level of strengthened capacity even if this wasn’t as actively facilitated as compared with the Ugandan partners. These considerations combined with the qualitative nature of the study design suggest care should be taken not to overgeneralise the study findings when considered in isolation. Study participants may also have been reluctant to express criticism of the Tiny Target programme given it was a partial source of income for many and given the primary interviewer (JP) was an employee of LSTM and Tiny Target programme management staff were often present at interview.

In conclusion, our study highlights three key lessons that may inform the design of national capacity strengthening activities conducted within the context of vector control partnerships. Adhering to the aforementioned recommendations derived from these lessons will increase the likelihood that: partnership members experience continuous capacity gains over the course of the partnership; capacities necessary for partnership activities are acquired; systemic constraints to greater independence on the part of the less well capacitated partners are addressed (at least in part); partnership activities benefit a wider cohort of individuals/organisations beyond those formally belonging to the partnership and that capacities gained during the course of the partnership are more likely to be sustained postpartnership. The generic nature of these recommendations suggests they are likely to be of benefit to many and diverse international partnerships within the wider global health space.

## supplementary material

10.1136/bmjph-2023-000410 online supplemental file 1

## Data Availability

Data are available upon reasonable request.

## References

[1] World Health Organisation (2023). Trypanosomiasis, human African (sleeping sickness). https://www.who.int/news-room/fact-sheets/detail/trypanosomiasis-human-african-(sleeping-sickness).

[2] Franco JR, Cecchi G, Paone M (2022). The elimination of human African trypanosomiasis: achievements in relation to WHO road map targets for 2020. PLoS Negl Trop Dis.

[3] Franco JR, Simarro PP, Diarra A (2014). Epidemiology of human African trypanosomiasis. Clin Epidemiol.

[4] World Health Organization (2012). Accelerating Work to Overcome the Global Impact of Neglected Tropical Diseases: A Roadmap for Implementation.

[5] Franco JR, Cecchi G, Priotto G (2017). Monitoring the elimination of human African trypanosomiasis: update to 2014. PLoS Negl Trop Dis.

[6] World Health Organisation (2020). Ending the Neglect to Attain the Sustainable Development Goals: A Road Map for Neglected Tropical Diseases 2021-2030.

[7] World Health Organisation (2013). Control and Surveillance of Human African Trypanosomiasis: Report of a WHO Expert Committee.

[8] Tirados I, Esterhuizen J, Kovacic V (2015). Tsetse control and Gambian sleeping sickness; implications for control strategy. PLoS Negl Trop Dis.

[9] Mahamat MH, Peka M, Rayaisse J-B (2017). Adding tsetse control to medical activities contributes to decreasing transmission of sleeping sickness in the Mandoul focus (Chad). PLoS Negl Trop Dis.

[10] Berté D, De Meeûs T, Kaba D (2019). Population genetics of Glossina palpalis palpalis in sleeping sickness foci of Cote D'Ivoire before and after vector control. Infect Genet Evol.

[11] Courtin F, Camara M, Rayaisse J-B (2015). Reducing human-Tsetse contact significantly enhances the efficacy of sleeping sickness active screening campaigns: a promising result in the context of elimination. PLoS Negl Trop Dis.

[12] Tirados I, Hope A, Selby R (2020). Impact of tiny targets on Glossina fuscipes quanzensis, the primary vector of human African trypanosomiasis in the democratic Republic of the Congo. PLoS Negl Trop Dis.

[13] Hope A, Mugenyi A, Esterhuizen J (2022). Scaling up of tsetse control to eliminate Gambian sleeping sickness in Northern Uganda. PLoS Negl Trop Dis.

[14] Bessell PR, Esterhuizen J, Lehane MJ (2021). Estimating the impact of tiny targets in reducing the incidence of Gambian sleeping sickness in the North-West Uganda focus. Parasit Vectors.

[15] Melachio Tanekou TT, Bouaka Tsakeng CU, Tirados I (2022). Environmental mutations in the Campo focus challenge elimination of sleeping sickness transmission in Cameroon. Med Vet Entomol.

[16] Ndung’u JM, Boulangé A, Picado A (2020). Trypa-NO! contributes to the elimination of gambiense human African trypanosomiasis by combining tsetse control with “screen, diagnose and treat” using innovative tools and strategies. PLoS Negl Trop Dis.

[17] World Health Organisation (2022). Benin, Uganda and Rwanda eliminate human African trypanosomiasis as a public health problem. https://www.who.int/news/item/24-05-2022-benin--uganda-and-rwanda-eliminate-human-african-trypanosomiasis-as-a-public-health-problem.

[18] Abomo P, Miaka EM, Crossman SJ (2021). Demonstrating the sustainability of capacity strengthening amidst COVID-19. Int Health.

[19] World Health Organisation (2017). Global Vector Control Response 2017-2030.

[20] Dean L, Gregorius S, Bates I (2017). Advancing the science of health research capacity strengthening in low-income and middle-income countries: a scoping review of the published literature, 2000-2016. BMJ Open.

[21] ESSENCE on Health Research (2016). Planning, Monitoring and Evaluation Framework for Research Capacity Strengthening.

[22] Mirzoev T, Topp SM, Afifi RA (2022). Conceptual framework for systemic capacity strengthening for health policy and systems research. BMJ Glob Health.

[23] Redman-MacLaren M, MacLaren DJ, Harrington H (2012). Mutual research capacity strengthening: a qualitative study of two-way partnerships in public health research. Int J Equity Health.

[24] Mormina M, Istratii R (2021). ‘Capacity for what? Capacity for whom?’ A decolonial deconstruction of research capcity development practices in the global South and proposal for a value-centred approach. Wellcome Open Res.

[25] Bates I, Boyd A, Smith H (2014). A practical and systematic approach to organisational capacity strengthening for research in the health sector in Africa. Health Res Policy Syst.

[26] Thomas DR (2006). A general Inductive approach for analyzing qualitative evaluation data. Am J Eval.

[27] Biesma RG, Brugha R, Harmer A (2009). The effects of global health initiatives on country health systems: a review of the evidence from HIV/AIDS control. Health Policy Plan.

[28] Haregu TN, Byrnes A, Singh K (2019). A Scoping review of non-communicable disease research capacity strengthening initiatives in low and middle-income countries. *Glob Health Res Policy*.

[29] Burgess HE, Chataway J (2021). The importance of mentorship and collaboration for scientific capacity-building and capacity-sharing: perspectives of African scientists. F1000Res.

[30] Aiyenigba A, Abomo P, Wiltgen Georgi N (2022). Enabling research capacity strengthening within a consortium context: a qualitative study. BMJ Glob Health.

[31] The Royal Society (2022). Royal society-FCDO Africa capacity building initiative (ACBI): lessons in facilitating wider access to consortia resources.

[32] Larson CP, Plamondon KM, Dubent L (2022). The equity tool for valuing global health partnerships. Glob Health Sci Pract.

