# Supplementary material for: National capacity strengthening within the context of an international vector control partnership: findings from a qualitative study conducted within the Ugandan ‘Tiny Targets’ programme
Source: BMJ Public Health. 2024 Apr 22;2(1):e000410. doi: 10.1136/bmjph-2023-000410 (PMC11812779; doi:10.1136/bmjph-2023-000410)
Supplement: online supplemental file 1 [file bmjph-2-1-s001.pdf]

INTERVIEW GUIDE

Study Title: Strengthening national capacity for tsetse control in Uganda

This is the full list of questions; however, individuals will only be asked a subset of questions dependent on their role and experience.

1. General Background

- What organisation do you currently work for?
- What is your role within the organisation?
  - Do you have any additional roles/work elsewhere?
- How long have you been in this (these) role(s)?
- What is the highest level of academic qualification you have obtained?
- Have you completed any specialist training to support you in your current role?

2. tsetse control activities/association

- Can you please describe your role on/association with the national tsetse control programme?
- How long have you been involved with the national tsetse control programme?
- Have you received any specialist training to support your role/association with the tsetse control programme?

3. tsetse control STRATEGY

- Do you have a national/district tsetse control strategic plan?
- What are the main themes/components of the strategy?
- How was the tsetse control strategy developed?
  - Who was involved in this process?
  - When was it developed?
- What role did local evidence play in the development of the national/district tsetse control strategy?

4. tsetse control implementation

- What organisations/individuals are involved in implementing the national tsetse control programme? (national and international)
- What are their various roles and what is the reporting structure?
- Is the national/district tsetse control programme integrated with other disease control/health programmes? In what way are they integrated?
- Are there any mechanisms to support public-private partnership on national tsetse control?
- How is the national tsetse control programme funded?
- How secure/stable has this funding been? Is it sufficient?
- Are there any foreseeable threats to current funding or opportunities for additional funding?

5. tsetse control monitoring, evaluation & research

- Is there a monitoring and evaluation (M&E) plan for the national/district tsetse control programme? If yes, please describe
- Who is responsible for implementing this M&E plan?
- How frequently are M&E results reported? And in what manner?
- Are there any research activities completed in support of the tsetse control programme? If yes, who is involved in research? How are research questions identified? How is research funded? And how are results communicated to the national/district tsetse control programme?

4. tsetse control infrastructure

- What physical resources are available to support the national tsetse control programme/your tsetse control activities?
- Where were these obtained? Are they readily accessible? Are they sufficient? If not sufficient, what else is needed?
- How are resources procured when needed?
  - How well does the procurement system work in practice?
    - What are the difficulties experienced?
    - What are potential solutions?

6. Training Activities for tsetse control

- How are training needs for tsetse control staff identified? Once identified, how are training needs addressed?
- What training opportunities are available for tsetse control staff?
- Have you attended any such training sessions? If so what impact has it had on your work?
- How are training opportunities identified and funded? Is there a core budget for training and how is it allocated?

7. challenges in tsetse control

- What are the biggest challenges you face in terms of completing your role on the national/district tsetse control programme? Or what are the biggest challenges facing tsetse control at the national/district level?
- How have you resolved these challenges? Or what is needed to resolve these challenges?

7. successes in tsetse control

- What have been the biggest successes in terms of tsetse control at the national/district level?
- What have been the most important factors contributing to this success?
- What would you like the national/district tsetse control programme to achieve within the next five years?
- What would be needed to meet these achievements?

7. other questions

- Are there any other comments you would like to make in regard to the national/district tsetse control programme?
- Do you have any questions you would like to ask me before completing this interview?
